# Automated Evaluation of Parapapillary Choroidal Microvasculature in Ischemic Optic Neuropathy and Open Angle Glaucoma

**DOI:** 10.1167/iovs.61.3.35

**Published:** 2020-03-19

**Authors:** Masoud Aghsaei Fard, Mirataollah Salabati, Raziyeh Mahmoudzadeh, Rahele Kafieh, Sahar Hojati, Mona Safizadeh, Sasan Moghimi, Robert Ritch, Prem S. Subramanian

**Affiliations:** 1 Farabi Eye Hospital, Tehran University of Medical Science, Tehran, Iran; 2 Medical Image and Signal Processing Research Center, School of Advanced Technologies in Medicine, Isfahan University of Medical Sciences, Isfahan, Iran; 3 Hamilton Glaucoma Center, Shiley Eye Center and Department of Ophthalmology, University of California, San Diego, La Jolla, California, United States; 4 Einhorn Clinical Research Center, New York Eye and Ear Infirmary of Mount Sinai, New York, New York, United States; 5 Sue Anschutz-Rodgers UC Health Eye Center, University of Colorado Hospital and Departments of Ophthalmology, Neurology, and Neurosurgery, University of Colorado School of Medicine, Aurora, Colorado, United States

**Keywords:** choroidal microvasculature, ischemic optic neuropathy, open angle glaucoma

## Abstract

**Purpose:**

To determine whether parapapillary choroidal microvasculature (PPCMv) density as measured by optical coherence tomography angiography differs between nonarteritic anterior ischemic optic neuropathy (NAION) and primary open angle glaucoma (POAG).

**Methods:**

Thirty-seven eyes with chronic NAION, 34 unaffected fellow eyes with NAION, 47 moderate and severe POAG eyes, and 54 healthy control subjects were evaluated. Automated PPCMv density was calculated using custom Matlab software in inner and outer annuli around the optic nerve region in addition to peripapillary superficial retinal vessels.

**Results:**

Linear models showed no difference in peripapillary retinal nerve fiber layer between NAION and POAG eyes. Mean peripapillary superficial small vessels in the NAION and POAG groups were 36.62 ± 7.1% and 39.72 ± 8.18% without a statistically difference between them (*P* = 0.16). Mean inner and outer annular region PPCMv densities in the NAION group were 26.55 ± 9.2% and 17.81 ± 6.9%, which were not different from unaffected fellow eyes and the control group. However, the POAG group had significantly reduced PPCMv density in both inner and outer annuli with values of 15.84 ± 6.5% and 12.80 ± 5.0%, respectively, compared with normal subjects (both *P* < 0.001). Inner and outer circle PPCMv densities were also significantly reduced in the POAG group compared with the NAION group.

**Conclusions:**

Reduced PPCMv density in POAG eyes shows that deep optic nerve head ocular blood flow may contribute to axonal damage in patients with glaucoma.

Both nonarteritic anterior ischemic optic neuropathy (NAION) and primary open angle glaucoma (POAG) are common optic neuropathies that manifest with peripapillary retinal nerve fiber layer (RNFL) and macular ganglion cell complex (GCC) damage.^1^ Optic nerve head blood flow abnormalities have also been found in these two diseases. Short posterior ciliary artery occlusion in NAION is associated with retrolaminar regional ischemia of the optic nerve head.^2^ Although mechanistically different, reduced choroidal flow to the laminar and prelaminar portions of the optic nerve (which are perfused by short ciliary arteries) has been suggested as one potential pathogenic mechanism of POAG.^3^^,^^4^ Therefore, evaluation of the deep optic nerve and choroidal microvasculature in these two optic neuropathies, which is feasible with optical coherence tomography angiography (OCT) or OCT-angiography (OCT-A), is of particular interest.

Peripapillary choroidal thickness as an indirect measure of choroidal vascularity has previously been measured in POAG and NAION. Of note, OCT showed a thinner peripapillary choroid in POAG in some studies^5^ and a thicker peripapillary choroid in NAION^6^ compared with control eyes. Additionally, OCT-A studies have shown choroidal microvascular dropout immediately around the optic disc in glaucomatous eyes using manual assessment.^7^^–^^11^ Choriocapillaris flow also was reported to be qualitatively impaired in NAION eyes studied by OCT-A,^12^ although another study did not find such changes.^13^ We used a new automated image processing method of choroidal microvessel quantification and compared the parapapillary choroidal microvascular changes in remote NAION and POAG to elucidate their roles in the pathogenesis of these two diseases.

## Methods

This comparative cross-sectional study was conducted from July 2016 to July 2018 in Farabi Eye Hospital. The study protocol was approved by the Institutional Review Board of Tehran University of Medical Science in accordance with the guidelines of the Declaration of Helsinki. Written informed consent was obtained from all participants. Participants with age ≥18 years, a spherical refraction within ±5.0 diopters, and cylinder correction within ±3.0 diopters were included. For all subjects, a complete ophthalmologic examination including best corrected visual acuity, slit-lamp biomicroscopy, intraocular pressure measurement using Goldmann tonometry, and axial length measurement (IOL Master; Carl Zeiss Meditec) were performed. Subjects then underwent OCT and OCT-A imaging using the AngioVue imaging system (Optovue, Inc., Fremont, CA, USA; RTVue XR, version 2018.0.0.18).

Three major groups of subjects were defined in this study:

1. Remote NAION group: Patients with a history of sudden, painless visual loss in one or both eyes >6 months before enrollment, and previous optic disc swelling and/or superficial hemorrhage typical of NAION that had resolved at the time of the study. Patients having or suspected of having an ocular or neurologic disease other than NAION, including but not limited to suspected glaucoma, acute NAION, arteritic AION, and inflammatory optic neuritis were excluded from this study. The data from healthy fellow eyes of these patients were also analyzed separately from the control group.
2. POAG group: Patients with enlarged vertical cup-to-disc ratio, diffuse or focal thinning of the neuroretinal rim, an open iridocorneal angle on gonioscopy, presence of a pattern standard deviation outside 95% normal limits (confirmed on at least two consecutive, reliable tests), and a glaucoma hemifield test outside normal limits were used for glaucoma diagnosis.^14^^,^^15^ Moderate and severe POAG eyes with Humphrey 24-2 mean deviation of less than -6 dB were recruited, so that the glaucoma and NAION groups would be similar in terms of severity.^1^^,^^14^^,^^15^ Presence or absence of peripapillary atrophy was not an inclusion or exclusion criterion.
3. Control group: This group included age-matched subjects with a best-corrected visual acuity ≥20/30, intraocular pressure <21 mm Hg, an open angle, normal optic disc appearance on fundus examination, and no visual field defects.

## Spectral-Domain Optical Coherence Tomography and Optical Coherence Tomography Angiography

All subjects underwent OCT and OCT-A imaging using the AngioVue imaging system. A standard circular scan was used to measure RNFL thickness and the mean and each sector RNFL values were recorded. The macular OCT was also performed to measure GCC thickness over a 7-mm diameter. Total, superior, and inferior hemispheric GCC were recorded. For all OCT imaging, scan dimensions were not adjusted for axial length.

For OCT-A, we used blood flow information at the radial peripapillary capillary (RPC) layer as a vessel density map (%) in a 4.5 × 4.5 mm rectangle scan centered on the optic disc. Both all-vessel and small-vessel densities were measured in whole-image and peripapillary areas and their superior and inferior sectors. Then, we used en face imaging of the choriocapillaris and employed customized MATLAB software (The MathWorks, Inc., Natick, MA, USA) for calculating a customized parapapillary choroidal microvasculature density after removing the shadows of the large retinal vessels and ignoring the information inside the disc.

**Figure 1.**
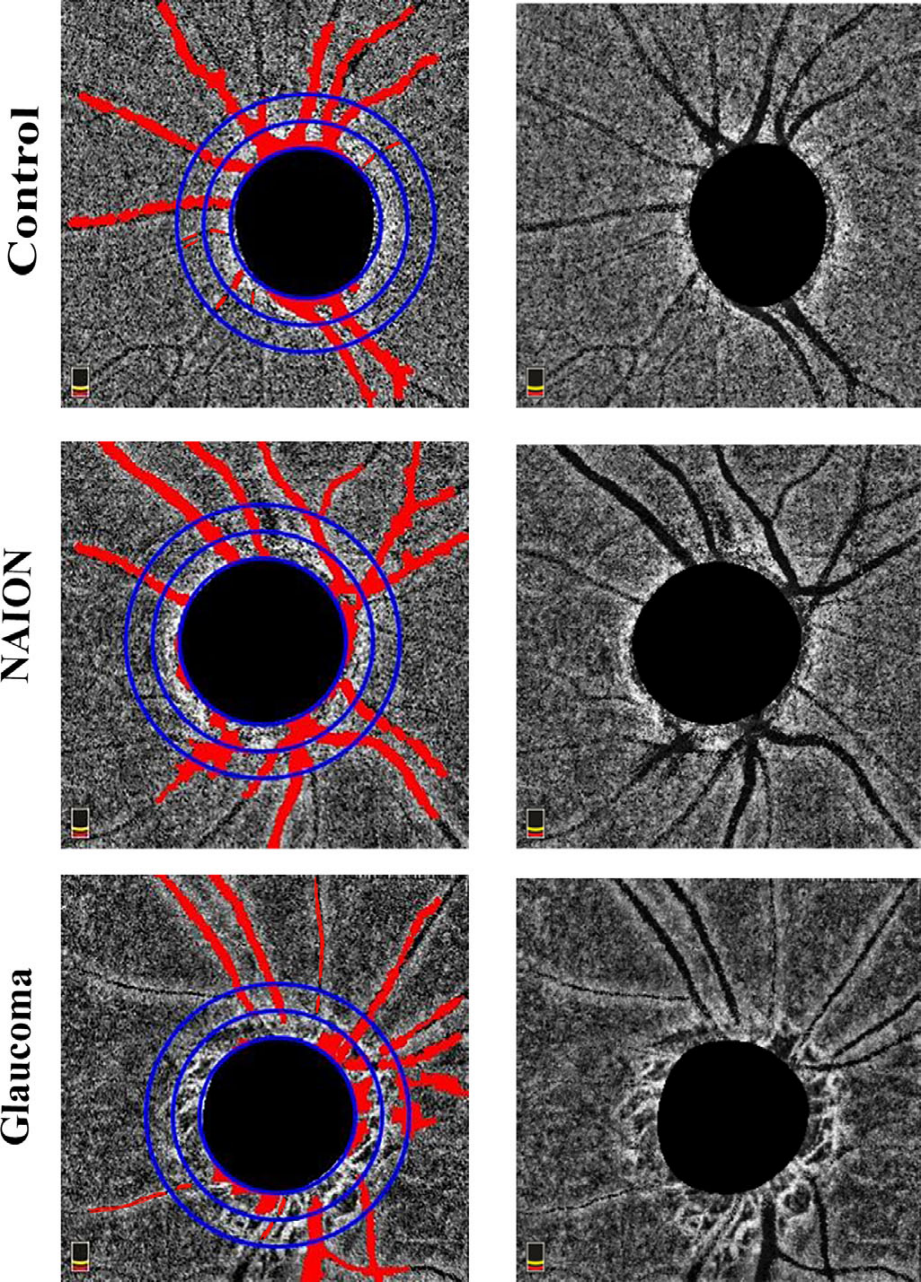
Parapapillary choroidal microvasculature in control, nonarteritic anterior ischemic optic neuropathy (NAION), and glaucoma subjects. Vessel density values of inner circle in control, NAION, and glaucoma eyes were 23.7%, 30.0%, and 17.7%.

Three concentric circles were overlaid on the en face image (Fig. 1). The inner circle was automatically circumscribed around the disc location, which was marked in black by the optovue OCT-A and had different diameters among images. Second, the middle circle and, last, the third circles were placed homocentrically with diameters of 1 mm and 2 mm greater than the inner circle, respectively. The information in the inner and outer coaxial annular regions of interest (ROI) with widths of 0.5 mm and their superior and inferior halves were then used for reporting parapapillary choroidal microvasculature (PPCMv) density values in populations. The main novelty in our calculation of PPCMv density is the automatic removal of black shadows because of large retinal vessels (which may be wrongly interpreted as black background region).

The customized PPCMv density algorithm consists of three main stages: Constructing the binary location of large retinal vessel shadows; parapapillary capillary segmentation using modified Otsu algorithm; and calculating PPCMv density values. In the first stage, to find the location of black shadows, a preprocessing stage was used to improve the image quality by histogram equalization and low-pass filtering. Then, a locally adaptive image thresholding was designed to calculate local first-order image statistics around each pixel, and a binary image with location of large retinal vessel shadows was constructed (union of all white locations as shadow vessels is called *U*). In the second stage, the location of parapapillary capillaries was determined by applying a modified Otsu algorithm on enface OCT-A images. In our modified Otsu algorithm, the pixels belonging to large vessel shadow locations (calculated as a binary image) were expurgated from Otsu calculations. The threshold value was also tested on macular en face OCTAs from the same device to ensure reliability. As a result, another binary image for location of parapapillary capillary was constructed and named *C*.

In the final stage, PPCMv density was calculated by dividing the sum of parapapillary capillary pixels on portions of en face OCTA images which did not contain any shadows from large retinal vessels. Equation (1) elaborates the PPCMv density formula to calculate the percentage of capillaries (*C*) on intersection of en face OCTA area (*E*) with complement on union of shadow vessels (*U*).

(1) $$CPVD=\;\frac{Area_{white}\;\left(C\right)}{Area\left(E-\;U\right)}=\;\frac{Area_{white}\;\left(C\right)}{Area\left(E\;\cap \;U^{c}\right)}$$

where *Area* is the number of pixels in an image, *Area_white_* is the number of pixels occupied with white pixels in a binary image, *E* is enface OCTA image, and  ^*c*^ stands for complement operator. To calculate PPCMv density in inner and outer coaxial annular ROIs and their superior and inferior halves, the *Area* operator was calculated only in desired geometrical partitions.

**Figure 2.**
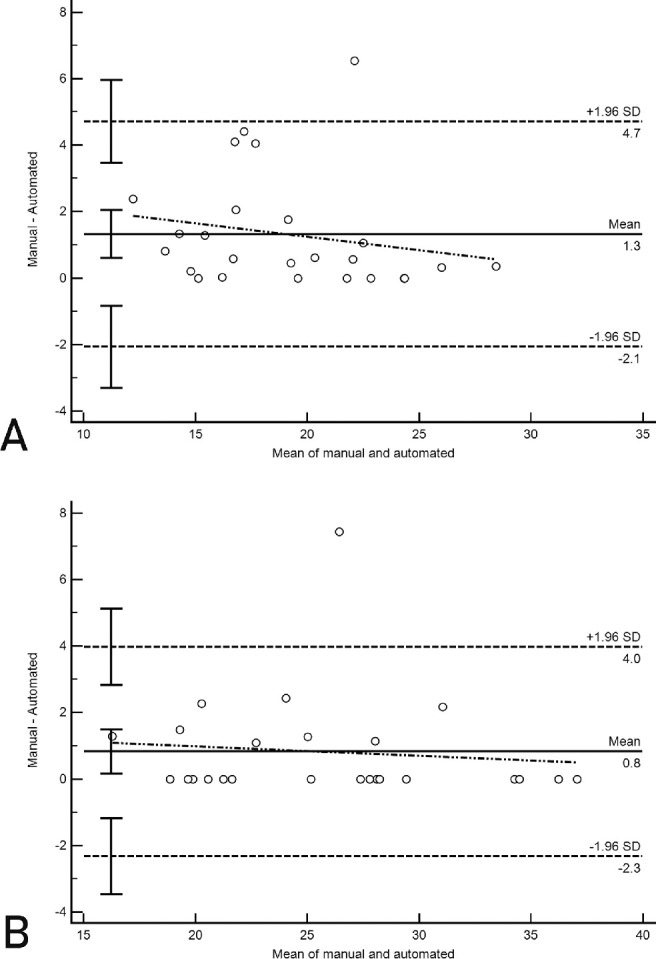
Bland-Altman plot of manual and automated parapapillary vessel measurements of (**A**) outer annulus and (**B**) inner annulus. Solid horizontal line: mean difference; dashed horizontal lines, 95% limits of agreement with 95% confidence intervals. Oblique dashed line shows the correlation between the mean difference and the magnitude of measurements.

Finally, to validate the performance of shadow vessel localization, manual labeling of large shadows was performed on 25 random images, and PPCMv density in inner and outer coaxial annular ROIs were compared between the proposed method and manual results by Bland-Altman plots (Fig. 2).

## Statistical Analysis

Descriptive statistics were calculated as the mean and standard deviation for normally distributed variables. Categorical variables were compared using the chi-squared test. We used a linear mixed model to evaluate the differences of peripapillary choroidal microvasculature density vessel density, RNFL, GCC, and RPC parameters between groups after accounting for inter-eye correlation and adjusting for multiple comparisons with Bonferroni correction.

Pearson correlation analysis was performed to assess the correlation of average RNFL thickness and vessel density of superficial and choroidal vessels in all study eyes. Associations between vessel density and average RNFL thickness were evaluated with univariate linear regression analysis.

Statistical analysis was performed with the SPSS software (IBM Corp. Released 2013. IBM SPSS Statistics for Windows, Version 22.0. Armonk, New York.). *P* values <0.05 were considered significant.

## Results

A total of 172 eyes of 94 subjects were enrolled in this study after excluding 5 eyes from the remote NAION group, 4 eyes from the NAION fellow eye group, 7 eyes from the POAG group, and 6 eyes from the control group because of poor image quality, retinal abnormalities, and/or eye movement. Therefore, 37 eyes of 36 patients with remote NAION, 34 unaffected fellow eyes of the NAION group, 47 eyes of 28 POAG group subjects, and 54 eyes of 30 healthy control subjects were used for analysis.

Table 1 shows demographic data and structural characteristics of study subjects. The differences of age, gender, and axial length between all study groups were not statistically significant. There were no significant differences in RNFL and GCC thicknesses between POAG and NAION groups; therefore, both NAION and POAG groups were matched for the degree of optic nerve and macular damage. Mean visual field index of POAG eyes was 77.1% (range, 7% to 99%).

**Table 1.** Demographic Features and Structural Data (Mean ± SD) in 4 Subject Groups

	Affected AION	Fellow AION	Glaucoma	Normal	AF	AN	GN	AG
Visual acuity (logMAR)	0.98 ± 1.01	0.19 ± 0.46	0.28 ± 0.37	0.03 ± 0.09	<0.001	<0.001	0.22	<0.001
Age (y)	55.46 ± 11.38	54.68 ± 11.29	60.38 ± 12.27	55.26 ± 15.69	0.78	>0.99	>0.99	0.95
Gender (male:female)	21:16	17:17	27:20	22:32	0.31			
Axial length (mm)	22.7 ± 0.88	22.96 ± 0.91	23.16 ± 0.89	22.8 ± 1.0	>0.99	>0.99	0.27	0.29
Total GCC (µm)	75.04 ± 15.0	94.98 ± 8.4	75.54 ± 21.2	99.09 ± 5.7	<0.001	<0.001	<0.001	>0.99
Superior GCC (µm)	74.18 ± 16.5	93.83 ± 9.0	77.70 ± 22.3	98.83 ± 6.1	0.001	<0.001	0.001	>0.99
Inferior GCC (µm)	75.94 ± 14.2	96.13 ± 8.2	72.01 ± 21.02	99.21 ± 5.7	<0.001	<0.001	<0.001	>0.99
Average RNFL (µm)	77.03 ± 23.4	108.85 ± 22.0	76.07 ± 15.4	109.19 ± 12.3	<0.001	<0.001	<0.001	>0.99
Superior RNFL (µm)	74.89 ± 23.5	108.21 ± 25.6	79.43 ± 18.1	110.89 ± 13.8	<0.001	<0.001	<0.001	>0.99
Inferior RNFL (µm)	79.94 ± 27.9	109.53 ± 21.3	74.04 ± 15.3	103.74 ± 21.4	<0.001	<0.001	<0.001	>0.99

Comparisons are based on linear mixed model analysis.

AF, Comparison between AION (anterior ischemic optic neuropathy) and fellow eye; AG, comparison between AION and glaucoma; AN, comparison between AION and normal subjects; FN, comparison between fellow eye and normal subjects; GCC, ganglion cell complex; GN, comparison between glaucoma and normal subjects; RNFL, retinal nerve fiber layer.

## OCT-A Results

Small and all vessel densities of the RPC layer (within RNFL layer) were compared between groups using linear mixed models in the peripapillary area and its superior and inferior hemifields. Both small and all vessel densities were significantly reduced in NAION and POAG patients compared with normal subjects (all *P* < 0.001). We found densities of 36.62 ± 7.1% and 43.99 ± 6.8% in the NAION group versus 51.74 ± 2.71% and 58.38 ± 2.5% in controls, respectively (both *P* < 0.001). The POAG group also showed lower mean peripapillary small and all vessels densities with value of 39.72 ± 8.18% and 46.45 ± 7.78 (both *P* < 0.001 vs. controls). There was no difference in peripapillary vessel density between NAION and POAG eyes (Table 2).

**Table 2.** Peripapillary Superficial Vessel Density (Mean ± SD) in 4 Subject Groups

Superficial Vessel Density (%)	AION	Fellow AION	Glaucoma	Normal	AF	AN	GN	AG
Peripapillary small vessels	36.62 ± 7.15	49.38 ± 5.66	39.72 ± 8.18	51.74 ± 2.71	<0.001	<0.001	<0.001	0.164
Peripapillary all vessels	43.99 ± 6.89	56.05± 5.66	46.45 ± 7.78	58.38 ± 2.57	<0.001	<0.001	<0.001	0.420
Superior small vessels	35.93 ± 8.51	48.70 ± 6.29	40.26 ± 8.87	51.81 ± 2.93	<0.001	<0.001	<0.001	0.040
Superior all vessels	43.41 ± 8.03	55.69 ± 6.32	47.22 ± 8.56	58.59 ± 2.77	<0.001	<0.001	<0.001	0.075
Inferior small vessel	37.64 ± 7.73	50.08 ± 5.73	38.80 ± 8.02	51.65 ± 2.89	<0.001	<0.001	<0.001	>0.99
Inferior all vessels	44.65 ± 6.79	55.65 ± 5.46	45.58 ± 7.67	57.99 ± 2.66	<0.001	<0.001	<0.001	>0.99

Comparisons are based on linear mixed model analysis.

AF, comparison between AION (anterior ischemic optic neuropathy) and fellow eye; AG, comparison between AION and glaucoma; AN, comparison between AION and normal subjects; FN, comparison between fellow eye and normal subjects; GN, comparison between glaucoma and normal subjects.

For validation of our automated customized PPCMv density, we first compared manual and automated methods of PPCMv density calculation using Bland-Altman plots (Fig. 2). The mean of differences (bias) between manual and automated measurements was small, and limits of agreement values, representing the range of values in which agreement between methods would lie for approximately 95% of the sample, were small. The bias was 1.3% for outer annulus PPCMv vessels (*P* = 0.28) and 0.8% for inner annulus PPCMv density (*P* = 0.62) by the two methods. Limits of agreement for the outer annular ring were -2.03% to 4.63%. The inner annular ring had limits of agreement from -2.33% to 3.93%.

Therefore, PPCMv density was measured in inner and outer annuli and inferior and superior hemifields around the optic nerve head by the automated method. Mean inner and outer annular vessel densities in the NAION group were 26.55 ± 9.2% and 17.81 ± 6.9%, which did not differ from either unaffected fellow eyes (27.52 ± 7.5% and 21.0 ± 5.9%; *P* > 0.99 and 0.06, respectively) or from controls (25.46 ± 6.6% and 19.15 ± 5.0%; both *P* > 0.99, respectively). However, the POAG group had significantly reduced PPCMv density in both inner and outer annuli with amounts of 15.84 ± 6.5% and 12.80 ± 5.0%, respectively, compared with normal controls (both *P* < 0.001) (Table 3). Further and even more important, inner and outer circle choroidal microvasculature densities were significantly reduced in the POAG group compared with the NAION group (*P* < 0.001 and *P* < 0.001, respectively). All outer and inner ring hemifields also had lower density in POAG than in NAION (Table 3).

**Table 3.** Parapapillary Choroidal Vasculature Density (Mean ± SD) in 2 Circles Around the Optic Nerve Head in 4 Subject Groups

Parapapillary Choroid, %	AION	Fellow AION	Glaucoma	Normal	AF	AN	GN	AG
Inner annulus	26.55 ± 9.2	27.52 ± 7.5	15.84 ± 6.5	25.46 ± 6.6	>0.99	>0.99	<0.001	<0.001
Inner hemi-superior	26.53 ± 9.1	28.13 ± 8.8	17.23 ± 7.8	26.14 ± 7.5	>0.99	>0.99	<0.001	<0.001
Inner hemi-inferior	26.56 ± 11.4	26.91 ± 9.3	14.45 ± 6.8	24.79 ± 7.5	>0.99	>0.99	<0.001	<0.001
Outer annulus	17.81 ± 6.9	21.0 ± 5.9	12.80 ± 5.0	19.15 ± 5.0	0.06	>0.99	<0.001	0.001
Outer hemi-superior	18.03 ± 7.4	21.34 ± 6.1	13.74 ± 5.8	19.79 ± 5.6	0.11	>0.99	<0.001	0.016
Outer hemi-inferior	17.60 ± 8.3	20.66 ± 8.0	11.87 ± 5.4	18.50 ± 5.9	0.21	>0.99	<0.001	0.003

Comparisons are based on linear mixed model analysis.

AF, comparison between AION (anterior ischemic optic neuropathy) and fellow eye; AG, comparison between AION and glaucoma; AN, comparison between AION and normal subjects; FN, comparison between fellow eye and normal subjects; GN, comparison between glaucoma and normal subjects.

Results from Pearson correlation analysis showed that mean peripapillary superficial small and all vessels (RPC layer) were significantly correlated with average RNFL thickness in all study eyes (*r* = 0.69, *P* < 0.001 and *r* = 0.71, *P* < 0.001, respectively). Univariate linear regression analysis showed that each 1-mm loss in RNFL thickness was associated with a 2.5% decrease in peripapillary superficial small vessels (*P* < 0.001). Although there were also correlations between average RNFL and inner and outer annuli PPCMv (*r* = 0.42, *P* < 0.001 and *r* = 0.53, *P* < 0.001, respectively), these correlations were not as strong as the correlation between RNFL and peripapillary superficial small and all vessels.

## Discussion

This study investigated differences in PPCMv densities in moderate and severe POAG eyes, NAION eyes, and healthy controls using automated OCT-A analysis. NAION and control eyes were not different from each other in PPCMv density. However, the POAG group had significantly reduced PPCMv density in both inner and outer annuli compared to normal subjects. Although parapapillary superficial vascular densities in the RNFL layer (both large and small vessels) were not different between NAION and POAG eyes matched for RNFL damage, PPCMv were statistically different between NAION and POAG. Correlations of RNFL with parapapillary superficial vessel density were stronger than correlations of RNFL with PPCMv density, although both were significant.

Many studies have recently reported the role of OCT-A in glaucoma and NAION. Indeed, we previously showed that both POAG and NAION eyes manifested similar peripapillary vessel density rarefaction in the superficial layer of the retina (RNFL), which correlated strongly with peripapillary RNFL thickness.^16^ Additionally similar involvement of superficial vessels in pale discs secondary to NAION and optic neuritis was shown.^17^ Therefore, it is evident that superficial vessel density loss might be secondary to RNFL damage and its decreased need for blood flow regardless of the cause of the optic neuropathy.^16^^,^^18^

However, the parapapillary microvasculature of the deep optic nerve and choroid may be involved differently in NAION and POAG. Several studies suggest that changes in choroidal microvasculature may represent a potential risk factor for POAG progression. Park et al.^10^ suggested that choroidal microvasculature focal loss is associated with progressive RNFL thinning and might affect the perfusion of the optic nerve. More important, baseline PPCMv within the β-zone parapapillary atrophy was associated with progression of glaucoma.^19^

In this study, we used an automated technique to measure parapapillary choroidal vessels in inner and outer annuli around the optic nerve; prior work used manual identification. In agreement with previous studies, we found focal loss of vessels in the inner and outer annuli in POAG eyes compared with control eyes.^10^^,^^11^^,^^19^ Although focal loss of vessels suggests that impairment in parapapillary choroidal perfusion may primarily cause vascular damage to the axons, it may be a simple secondary effect of glaucomatous damage. To address this question, we compared superficial (within RNFL) and deep vessel densities of two optic neuropathies (NAION and POAG) with similar RNFL loss, allowing us to evaluate the relationship between neural loss and vascular damage. We found similar superficial vessel loss in both conditions but more parapapillary choroidal vasculature loss in POAG than NAION eyes. This suggests that, although the superficial parapapillary vessel dropout could be secondary to microvascular changes from RNFL loss, reduced parapapillary deep choroidal vasculature in POAG eyes could contribute primarily to axonal damage in patients with glaucoma. In fact, if deep vessel loss was due to RNFL loss, we would expect to observe deep vessel loss in NAION eyes as well. Stronger correlation of RNFL with peripapillary superficial vessel density than correlation of RNFL with PPCMv density also suggests secondary superficial vessel dropout in contrast to primary PPCMv dropout. Similarly, Lee et al.^9^ proposed primary involvement of deep vessels in the pathogenesis of glaucoma. They speculated that because the choroidal microvasculature is not directly related to the prelaminar tissue supply in glaucoma, the choroidal vessels cannot be obliterated as a secondary change to the loss of prelaminar tissue. However, that speculation has been challenged by the idea of blood supply of prelaminar tissue from choroid in a case report of three glaucoma cases.^20^

In contrast to this scenario, PPCMv was spared in NAION, which demonstrates involvement of the only short posterior ciliary vessels without choroidal contribution in the pathogenesis of NAION. In fact, we previously showed that peripapillary choroidal thickness by enhanced depth imaging (EDI)-OCT is not thin in NAION but actually thicker in both affected fellow eyes than in control eyes^6^; this finding has been confirmed in other studies.^21^^,^^22^ We postulate that a thick choroid predisposes to NAION by increasing prelaminar neural tissue that might be compressed by the deeper PPCMv. We also previously showed thick prelaminar tissue using EDI-OCT in NAION eyes comprises a component of crowded optic discs in such eyes.^23^^,^^24^ It is possible that PPCMv may be *increased* in NAION, but OCT-A may lack sensitivity to identify such a change, if present. There may be insufficient resolution of compacted vessels in the tight parapapillary space. Additionally, prior OCT studies assessed total choroidal thickness, but this measurement cannot be compared directly with choroidal microvasculature density by OCT-A. Finally, the thick choroid might affect OCT-A measurements and signals in our study. However, we did not measure peripapillary choroidal thickness in this study with EDI-OCT, which is one of our study limitations. Future development of new segmentation methods, manual or automated, may improve our ability to measure these OCT-A parameters and allow us to more precisely evaluate the total choroidal vascular anatomy in NAION and normal eyes. In addition, considering the proposed method for calculation of PPCMv, two other limitations should be considered. The first issue relates to limited resolution of en face OCTA images, which results in some highly compact regions around the disk location. We did not exclude such regions from *C* set in current study. Because this issue could affect all images, different PPCMv density that was found in this study should not be artifactual. The second issue is the white artifacts around shadows from retinal vessels. Such artifacts are contrast differences mostly from vessels' shadows and should not be considered as white capillary data; we tried to localize them in this study, but they were mostly located outside the bigger concentric circle and thus did not affect the final result.

In conclusion, in NAION and POAG eyes with similar RNFL loss and consequently similar peripapillary superficial vascular densities dropout in RPC layer (both large and small vessels), PPCMv differed significantly. although inner annulus PPCMv density was lower in POAG eyes compared with NAION, outer annulus PPCMv was not different between NAION and POAG groups. This difference may reflect the varying roles that PPCMv plays in the two optic neuropathies, and further study is indicated to determine the pathogenic role that such changes may play.
